# lncRNA SNHG4 modulates colorectal cancer cell cycle and cell proliferation through regulating miR-590-3p/CDK1 axis

**DOI:** 10.18632/aging.202737

**Published:** 2021-03-19

**Authors:** Zhongyi Zhou, Fengbo Tan, Qian Pei, Chenglong Li, Yuan Zhou, Yuqiang Li, Haiping Pei

**Affiliations:** 1 Department of General Surgery, Xiangya Hospital, Central South University, Changsha 410008, Hunan, China; 2 National Clinical Research Center for Geriatric Disorders, Xiangya Hospital, Central South University, Changsha 410008, Hunan, China

**Keywords:** colorectal cancer (CRC), lncRNA SNHG4, miR-590-3p, CDK1, cell cycle

## Abstract

Colorectal cancer (CRC) is a prevalent malignancy worldwide. The development of genome sequencing technology has allowed the discovery that epigenetic regulation might play a critical role in CRC tumorigenesis. In the present study, we found that the long noncoding RNA (lncRNA) SNHG4 was dramatically increased in CRC tissue samples and cell lines based on both publicly available and experimental data. SNHG4 knockdown suppressed the viability and colony formation capacity of CRC cells. The expression of CDK1 was considerably increased in CRC tissue samples and cells and had a positive correlation with the expression of SNHG4 in CRC. SNHG4 silencing not only caused S phase cell cycle arrest but also significantly downregulated the CDK1, cyclin B1, and cyclin A2 protein levels in CRC cells. miR-590-3p simultaneously bound to SNHG4 and CDK1. miR-590-3p functioned to inhibit CDK1 expression. miR-590-3p overexpression exerted the same effects on the CRC cell phenotype as SNHG4 knockdown. The effects of si-SNHG4 on CRC cells were significantly reversed by anti-miR-590-3p, indicating that SNHG4 relieved the miR-590-3p-induced inhibition of CDK1 by acting as a competing endogenous RNA (ceRNA). *In vivo*, SNHG4 silencing inhibited subcutaneously transplanted tumor growth and decreased cell cycle marker levels, whereas miR-590-3p inhibition exerted the opposite effects. The *in vivo* effects of SNHG4 silencing were also reversed by miR-590-3p inhibition. The SNHG4/miR-590-3p/CDK1 axis influences the cell cycle to modulate CRC cell proliferation and subcutaneously transplanted tumor growth. Further application of this axis still requires analysis using more animal models and clinical investigations.

## INTRODUCTION

Colorectal cancer (CRC) is one of the most prevalent malignancies and the fourth highest mortality of all types of cancers worldwide [1, 2]. The high incidence and relapse rate, as well as the acquisition of resistance to chemotherapy, make CRC a substantial public health challenge. Despite the development of CRC therapies in recent years, the prognosis of patients with CRC is still unsatisfactory, with a poor 5-year survival rate [3]. Therefore, it is urgent to investigate the molecular mechanisms underlying the tumorigenesis and progression of CRC to develop better individualized treatments.

Genome sequencing technology studies and many related studies have indicated that most genome sequence transcripts are noncoding RNAs (ncRNAs), and the main members of this group are either long noncoding RNAs (lncRNAs) or microRNAs (miRNAs) [4–6]. In colorectal cancer, although the roles of miRNAs have long been recognized, the roles of lncRNAs as biological targets for diagnostic [7], prognostic [8], and therapeutic applications [9, 10] have only emerged in recent decades. For example, lncRNA colon cancer-associated transcript 2 (CCAT2) enhances the activity of Wnt signaling by increasing MYC, miR-17-5p, and miR-20a, ultimately leading to chromosomal instability and metastatic progression in colon cancer [11]. LncRNA cancer susceptibility candidate 2 (CASC2) increases protein inhibitor of activated signal transducer and activator of transcription 3 (PIAS3) by serving as a specific sponge and ceRNA for miR-18a, ultimately prolonging G0/G1-S phase transition, inhibiting CRC cell proliferation and suppressing tumor growth [12]. With the development of high-throughput sequencing and microarray techniques, increasing alterations in ncRNA profiling have been revealed [13]. In this context, the present study attempted to analyze publicly available data from TCGA colorectal adenocarcinoma database, Genotype-Tissue Expression (GTEx), and Gene Expression Omnibus (GEO) to identify lncRNAs that might participate in CRC development.

Regarding their molecular mechanisms, lncRNAs transcriptionally and posttranscriptionally affect gene expression, thus contributing to chromatin remodeling, RNA decay, epigenetic modulation, chromatin modification and many other cell functions [14, 15] and modulating tumor formation, invasion and metastasis [16, 17]. Recently, increasing evidence has indicated that lncRNAs can competitively bind to miRNAs via their MREs (miRNA response elements) to act as ceRNAs (competing endogenous RNAs), thereby regulating the expression of downstream target RNAs [18]. Thus, herein, we also examined publicly available microarray expression profiles to identify differentially expressed genes in CRC that were positively correlated with candidate lncRNAs and cooperated with lncRNAs to affect CRC progression in a miRNA-related manner. Moreover, the online tool mirDIP and the R programming language (based on the complementary model of 7mer+A or 8mer+A) predicted the miRNAs that could simultaneously bind to selected lncRNAs and mRNAs. The putative binding between selected miRNAs and lncRNAs/mRNAs were confirmed. The specific roles of lncRNAs and miRNAs in target mRNA expression and CRC cell phenotypes were examined individually and in combination. The correlations of the expression levels of these molecules in tissue samples were analyzed to confirm the existence of this lncRNA-miRNA-mRNA axis in CRC. Taken together, we described a new lncRNA-miRNA-mRNA axis and demonstrated its role and underlying mechanism in colorectal cancer.

## RESULTS

### Expression of lncRNA SNHG4 (long noncoding RNA SNHG4) based on publicly available and experimental data

**Figure 1:**
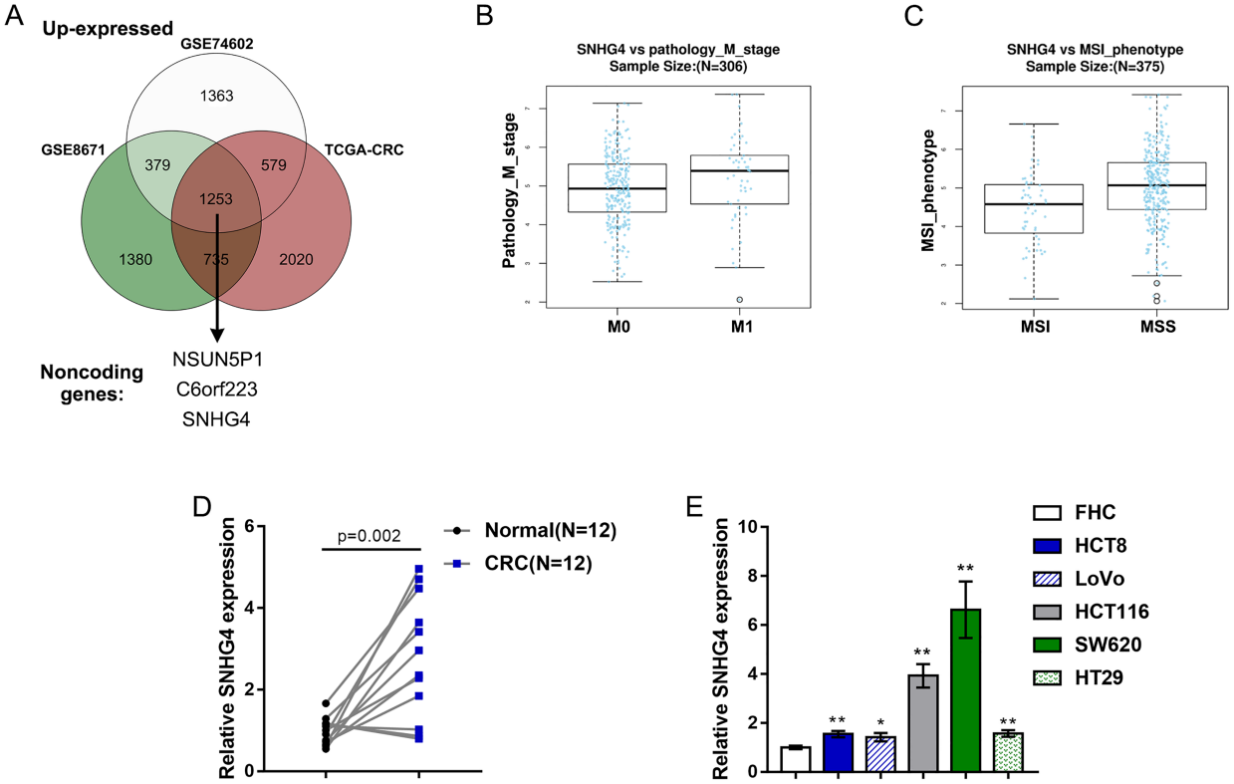
**Expression of long noncoding RNA SNHG4 (lncRNA SNHG4) based on publicly available and experimental data.** (**A**) The expression of SNHG4 in colorectal and normal noncancerous tissues according to the publicly available microarray expression profiles GSE8671, GSE74602, and TCGA Colon and Rectal Cancer (TCGA-CRC). (**B**) The expression of SNHG4 in colorectal cancer (CRC) M0-stage (nonmetastatic) and M1-stage (lymphatic or distal metastatic) tissues according to TCGA-CRC data. (**C**) The expression of SNHG4 in CRC microsatellite instability (MSI) and microsatellite stable (MSS) tissues according to TCGA-CRC data. (**D**) The expression of SNHG4 was determined in 12 CRC and normal noncancerous tissues by real-time PCR. (**E**) The expression of SNHG4 was determined in one normal fetal colon cell line (FHC) and five CRC cell lines (HCT8, LoVo, HCT116, SW620, and HT29) by real-time PCR. ^*^P < 0.05, ^**^P < 0.01, compared to FHC cells.

As mentioned above, we downloaded and analyzed three sets of publicly available microarray expression profiles, namely, the GSE8671, GSE74602, and TCGA Colon and Rectal Cancer (TCGA-CRC) datasets, to identify differentially upregulated lncRNAs in CRC. Through cross-check, we found that a total of 3 lncRNAs, including C6orf223, NSUN5P1 and SNHG4, were significantly upregulated (*p* < 0.05, log2FC > -0.56) in CRC (Figure 1A). The role of C6orf223 and NSUN5P1 in cancer has not yet been reported, but many studies have shown that SNHG4 is involved in multiple cancers, including osteosarcoma [19], prostate cancer [20], and cervical cancer [21]. Nevertheless, the biological function and underlying molecular mechanism of SNHG4 in CRC remain unclear and require further clarification. As further evidence, according to TCGA-CRC data, SNHG4 expression was dramatically increased in CRC M1-stage (lymphatic or distal metastatic) tissue samples compared with CRC M0-stage (nonmetastatic) tissue samples (Figure 1B) and was markedly enhanced in CRC microsatellite stable (MSS) tissue samples compared with CRC microsatellite instability (MSI) tissue samples (Figure 1C). The expression of SNHG4 was obviously increased in CRC tissues compared with normal noncancerous tissues (Figure 1D). Furthermore, SNHG4 expression was detected in one normal fetal colon cell line (FHC) and five CRC cell lines (HCT8, LoVo, HCT116, SW620, and HT29). The expression of SNHG4 was dramatically upregulated in the HCT116 and SW620 cell lines (Figure 1E); thus, the HCT116 and SW620 cell lines were selected for further experiments.

### Effects of SNHG4 on CRC cell proliferation and metastasis

**Figure 2:**
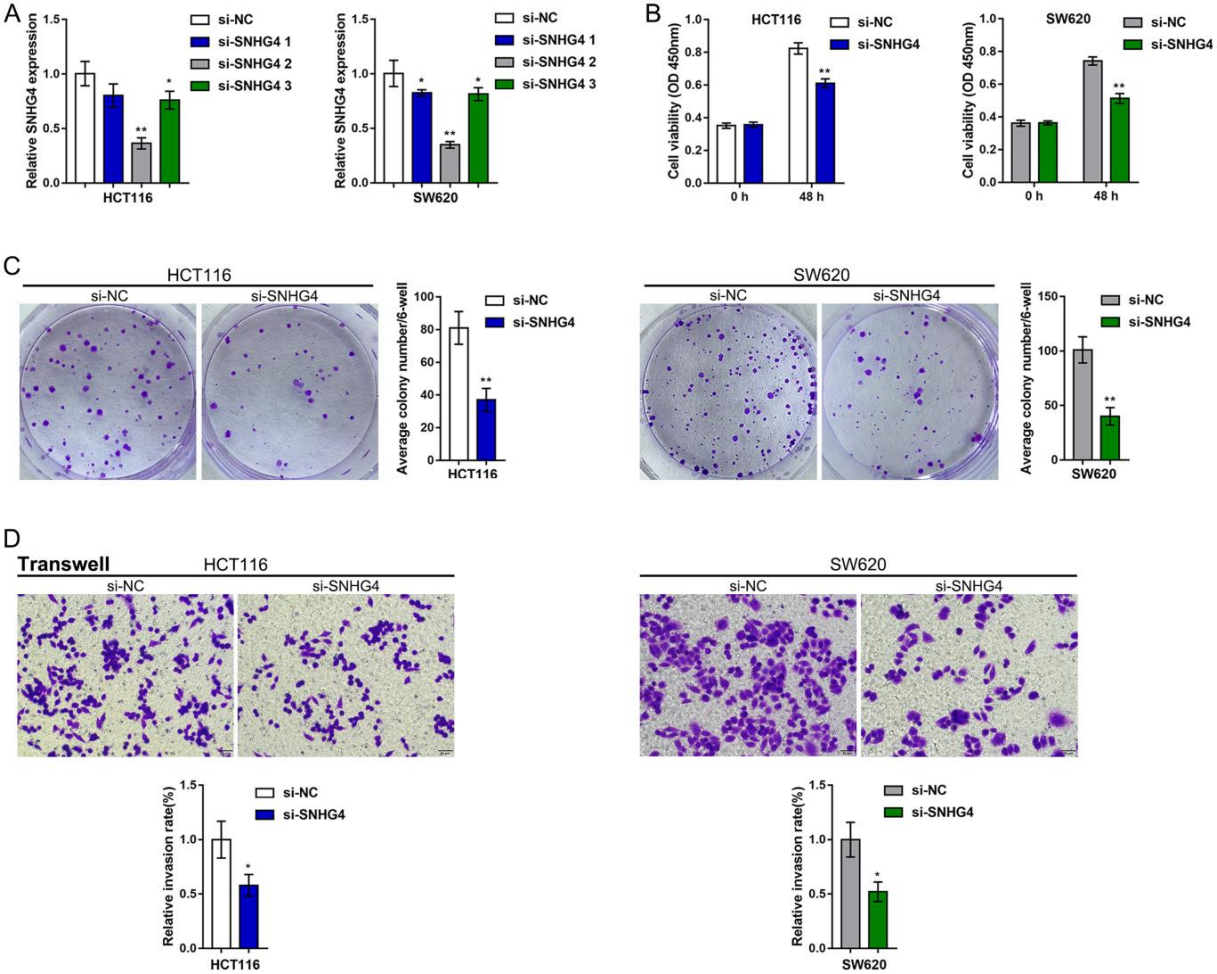
**Effects of SNHG4 on CRC cell proliferation and metastasis.** (**A**) SNHG4 was knocked down in HCT116 and SW620 cells by transfection of the cells with si-SNHG4 1/2/3. The transfection efficiency was validated by real-time PCR. Si-SNHG4 2 was selected for further experiments due to its better transfection efficiency. (**B–C**) HCT116 and SW620 cells were transfected with si-SNHG4, and (B) cell viability was examined by CCK-8 assay and (C) colony formation capacity was examined by colony formation assay. (**D**) HCT116 and SW620 cells were transfected with si-SNHG4, and cell invasion ability was examined by Transwell assay. ^*^P < 0.05, ^**^P < 0.01, compared to the si-NC group.

Since SNHG4 expression is significantly upregulated in CRC, to investigate its function, we transfected the HCT116 and SW620 cell lines with si-SNHG4 1/2/3 to knockdown SNHG4, and this knockdown was confirmed by real-time PCR. Then, we selected si-SNHG4 2 because of its higher transfection efficiency (Figure 2A). After knocking down SNHG4, HCT116 and SW620 cell viability and colony formation ability were significantly inhibited (Figure 2B–2C). Then, the effects of SNHG4 knockdown on CRC cell metastasis and microsatellite stability were detected. As shown in Figure 2D, silencing SNHG4 markedly inhibited HCT116 and SW620 cell invasion. The effect of SNHG4 on the expression of microsatellite stability-related proteins (MLH1, PMS2, MSH2, and MSH6) was also determined in SW620 cells (Supplementary Figure 1). Knockdown of SNHG4 markedly inhibited MLH1, PMS2, MSH2, and MSH6 protein expression. In summary, SNHG4 might act as an oncogene in CRC.

### SNHG4 is correlated with cyclin-dependent kinase 1 (CDK1) and the CRC cell cycle

**Figure 3:**
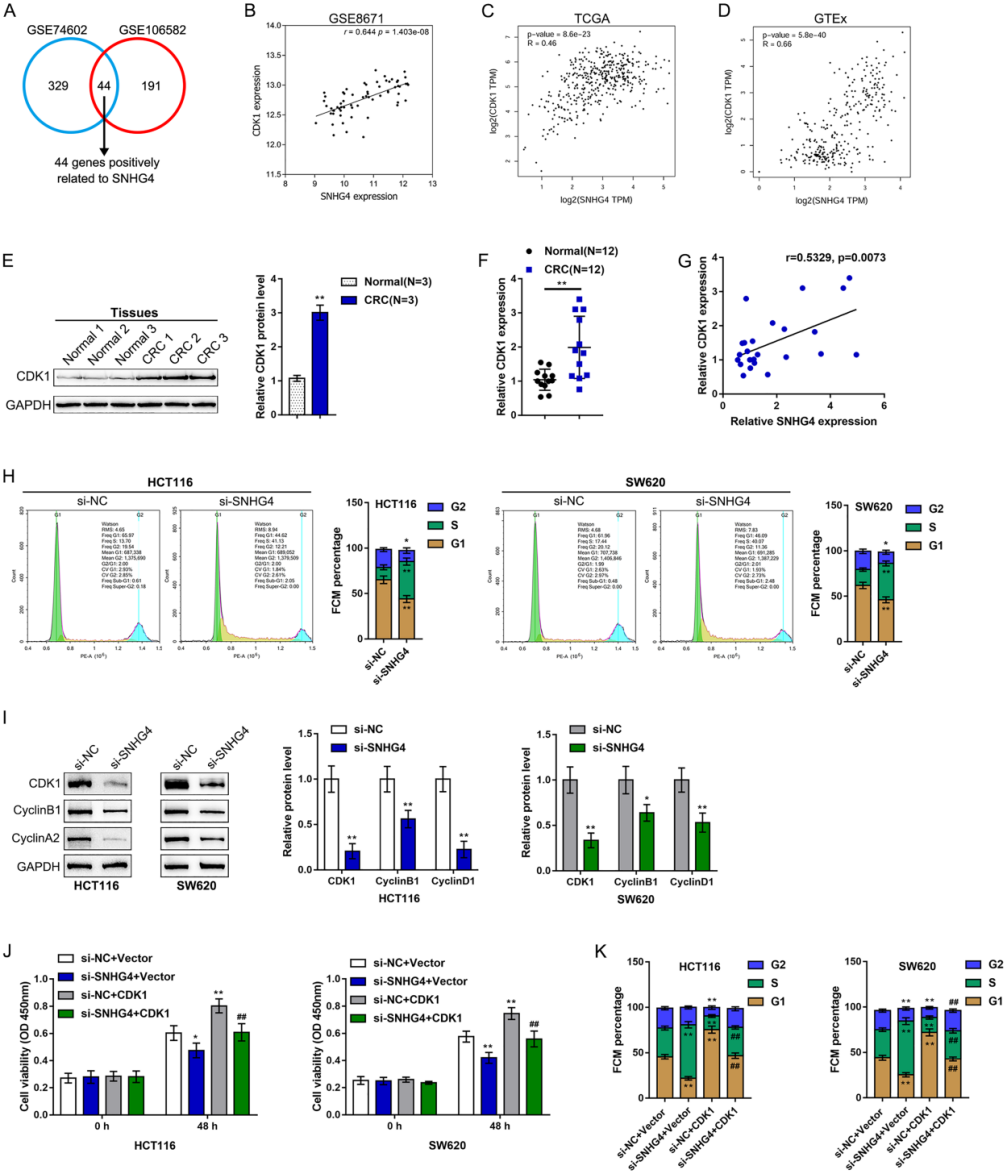
**SNHG4 is correlated with cyclin-dependent kinase 1 (CDK1) and the CRC cell cycle.** (**A**) Samples in GSE106582 and GSE74602 were divided into high SNHG4 expression and low SNHG4 expression groups. The correlation between SNHG4 and differentially expressed genes was analyzed to identify genes that are significantly positively correlated with SNHG4 (*r* > 0.40, *p* < 0.05); a total of 44 genes were found to be positively correlated with SNHG4 (SKA3, TOP1MT, RFC3, DSCC1, PPIL1, UBE2C, MND1, IPO4, C10orf2, CDK1, AUNIP, DDIAS, FEN1, HELLS, CDC45, PRR7, SPC25, GGCT, KIF14, RPL13, PRMT3, PROX1, CKAP2L, SLC39A10, HMMR, KIF4A, NOLC1, EXO1, MCM6, CCNF, SPDL1, NOP16, KIF20A, MCM10, EPHX4, MMP10, FJX1, NUF2, CBX2, NEK2, ANGPT2, ECT2, KIF23, and ESM1). (**B–D**) Correlation of the SNHG4 and CDK1 expression levels based on data from GSE8671, TCGA, and GTEx. (**E**) The protein levels of CDK1 in tissue samples as determined by immunoblotting. (**F**) The expression of CDK1 in tissue samples as determined by real-time PCR. (**G**) The correlation between the SNHG4 and CDK1 expression levels as determined by Pearson’s correlation analysis. (**H**) HCT116 and SW620 cells were transfected with si-SNHG4, and the cell cycle was examined by flow cytometry. (**I**) HCT116 and SW620 cells were transfected with si-SNHG4, and the protein levels of CDK1, cyclin B1, and cyclin A2 were examined. (**J**) HCT116 and SW620 cells were transfected with si-SNHG4 and/or CDK1 overexpression vector, and cell proliferation was examined by CCK-8 assay. (**K**) HCT116 and SW620 cells were transfected with si-SNHG4 and/or CDK1 overexpression vector, and cell cycle progression was examined by flow cytometry. ^*^P < 0.05, ^**^P < 0.01, ^##^*P* < 0.01.

To investigate the molecular mechanism, this study performed further analysis on publicly available microarray expression profiles. We divided the samples in GSE106582 and GSE74602 into high SNHG4 expression groups and low SNHG4 expression groups. The correlation between SNHG4 expression and differentially expressed gene expression was analyzed to identify genes with a significant positive correlation with SNHG4 (*r* > 0.40, *p* < 0.05). Forty-four genes, including SKA3, TOP1MT, RFC3, DSCC1, PPIL1, UBE2C, MND1, IPO4, C10orf2, CDK1, AUNIP, DDIAS, FEN1, HELLS, CDC45, PRR7, SPC25, GGCT, KIF14, RPL13, PRMT3, PROX1, CKAP2L, SLC39A10, HMMR, KIF4A, NOLC1, EXO1, MCM6, CCNF, SPDL1, NOP16, KIF20A, MCM10, EPHX4, MMP10, FJX1, NUF2, CBX2, NEK2, ANGPT2, ECT2, KIF23, and ESM1, were identified as being positively correlated with SNHG4 (Figure 3A). These 44 genes were subjected to KEGG annotation and GO (Gene Ontology) enrichment analyses to identify related signaling pathways. As shown in Supplementary Tables 1 and 2, KEGG annotation and GO enrichment analyses both showed that these 44 genes were significantly enriched in DNA replication, mismatch repair, and cell cycle pathways.

Of these 44 genes, CDK1, a kinase that acts on the core of the cell cycle [22, 23], attracted our attention. The CDK1/cyclin B1 complex not only mediates mitochondrial activities during cell cycle progression but also plays a critical role in tumor adaptive resistance [24–26]. Herein, according to publicly available GSE8671, TCGA, and GTEx data, the SNHG4 and CDK1 expression levels were significantly positively correlated (Figure 3B–3D). In collected tissue samples, both the CDK1 protein and mRNA expression levels were significantly increased in CRC tissues (Figure 3E–3F). As predicted by publicly available data, there was a positive correlation between the SNHG4 and CDK1 expression levels (Figure 3G).

Since CDK1 is a central factor in the cell cycle, SNHG4 knockdown was performed in HCT116 and SW620 cells, and the cell cycle was examined. As shown in Figure 3H, SNHG4 knockdown dramatically led to S phase cell cycle arrest. Moreover, SNHG4 knockdown markedly decreased the CDK1, cyclin B1, and cyclin A2 protein levels (Figure 3I). Moreover, we transfected si-SNHG4 and/or CDK1 overexpression vectors into HCT116 and SW620 cells to conduct rescue experiments. As shown in Figure 3J, SNHG4 knockdown markedly inhibited cell proliferation while CDK1 overexpression notably promoted cell proliferation in both HCT116 and SW620 cells; the activation effect of CDK1 on cell proliferation could be abolished by SNHG4 knockdown. Next, silencing SNHG4 clearly caused S phase cell cycle arrest, while CDK1 overexpression facilitated cell cycle progression (Figure 3K and Supplementary Figure 2). These data further suggest that SNHG4 might cross-talk with CDK1 to promote the proliferation of CRC cells by causing S phase cell cycle arrest.

### miR-590-3p binds to SNHG4 and CDK1 3'-UTR

**Figure 4:**
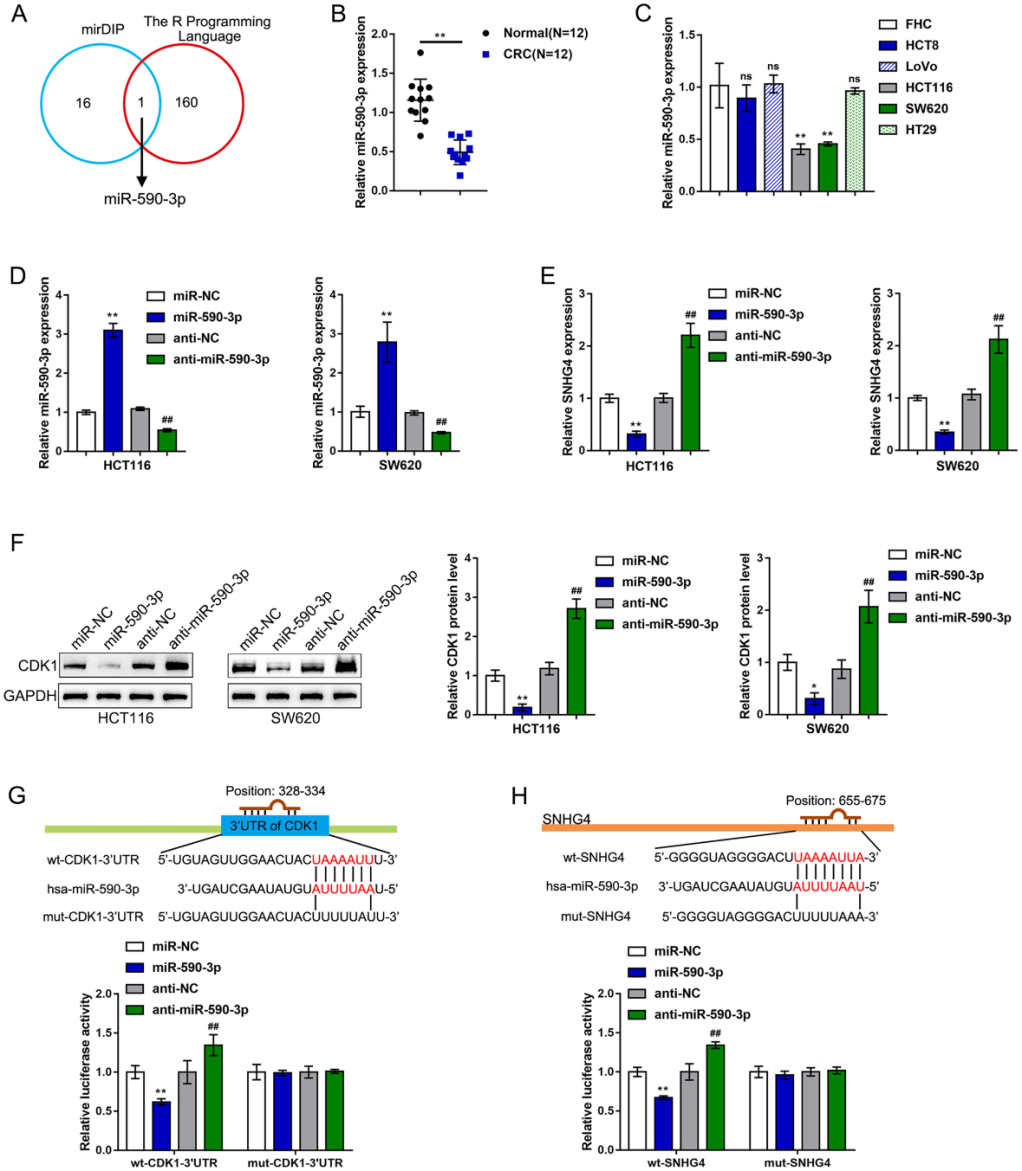
**miR-590-3p binds to SNHG4 and the CDK1 3'-UTR.** (**A**) The online tools mirDIP and R programming language were used to predict miRNAs that might simultaneously target SNHG4 and the CDK1 3'-UTR, and miR-590-3p was identified. (**B**) The expression of miR-590-3p was determined in 12 CRC and normal noncancerous tissues by real-time PCR. (**C**) The expression of miR-590-3p was determined in one normal fetal colon cell line (FHC) and five CRC cell lines (HCT8, LoVo, HCT116, SW620, and HT29) by real-time PCR. (**D**) miR-590-3p was overexpressed or inhibited in HCT116 and SW620 cells by transfection with miR-590-3p or anti-miR-590-3p, and the effects were confirmed by real-time PCR. (**E**) HCT116 and SW620 cells were transfected with miR-590-3p or anti-miR-590-3p, and the mRNA levels of SNHG4 were examined by real-time PCR. (**F**) HCT116 and SW620 cells were transfected with miR-590-3p or anti-miR-590-3p, and the protein levels of CDK1 were examined by immunoblotting. (**G–H**) Luciferase reporter assays were performed by constructing luciferase reporter vectors, as described in the Materials and methods section, to validate the predicted binding of miR-590-3p to SNHG4 and the CDK1 3'-UTR. ^*^P < 0.05, ^**^P < 0.01, ^##^*P* < 0.01.

It is widely accepted that lncRNAs can regulate the expression of target mRNAs downstream of miRNAs by interacting with these miRNAs [27, 28]. Herein, the online tool mirDIP and the R programming language (based on the complementary model of 7mer+A or 8mer+A) were used to predict miRNAs that might simultaneously target SNHG4 and the CDK1 3'-UTR, and miR-590-3p was identified (Figure 4A). In contrast to SNHG4 and CDK1 expression, miR-590-3p expression was markedly downregulated in 12 CRC tissue samples compared with normal noncancerous tissue samples (Figure 4B). In addition, miR-590-3p expression was significantly reduced in the CRC HCT116 and SW620 cell lines compared with the normal fetal colon cell line FHC (Figure 4C).

To validate the putative binding of miR-590-3p to SNHG4 and CDK1, we transfected HCT116 and SW620 cells with miR-590-3p or anti-miR-590-3p to establish miR-590-3p-overexpressing or miR-590-3p-inhibited cells; we performed real-time PCR to verify the transfection efficiency (Figure 4D). In both the HCT116 and SW620 cells, overexpression of miR-590-3p clearly suppressed, while knockdown of miR-590-3p clearly promoted, SNHG4 expression (Figure 4E). In the HCT116 and SW620 cell lines, the overexpression of miR-590-3p obviously downregulated, while the inhibition of miR-590-3p upregulated, the CDK1 protein levels (Figure 4F). Next, as described in the Materials and methods section, we constructed two different types of SNHG4 and CDK1 3'-UTR luciferase reporter vectors, namely, wild-type and mutant vectors. These reporter vectors were cotransfected into 293T cells with miR-590-3p or anti-miR-590-3p. The luciferase activity was then determined in each group. The overexpression of miR-590-3p remarkably downregulated, whereas the inhibition of miR-590-3p upregulated, the wt-SNHG4 or wt-CDK1 3'-UTR vector luciferase activities; mutating the putative miR-590-3p-binding site in the mut-SNHG4 or mut-CDK1 3'-UTR eliminated the changes in the luciferase activity (Figure 4G–4H). In summary, miR-590-3p binds to the CDK1 3'-UTR to inhibit CDK1 mRNA expression and decrease CDK1 protein levels.

### Effects of miR-590-3p on CRC cell proliferation and the cell cycle

**Figure 5:**
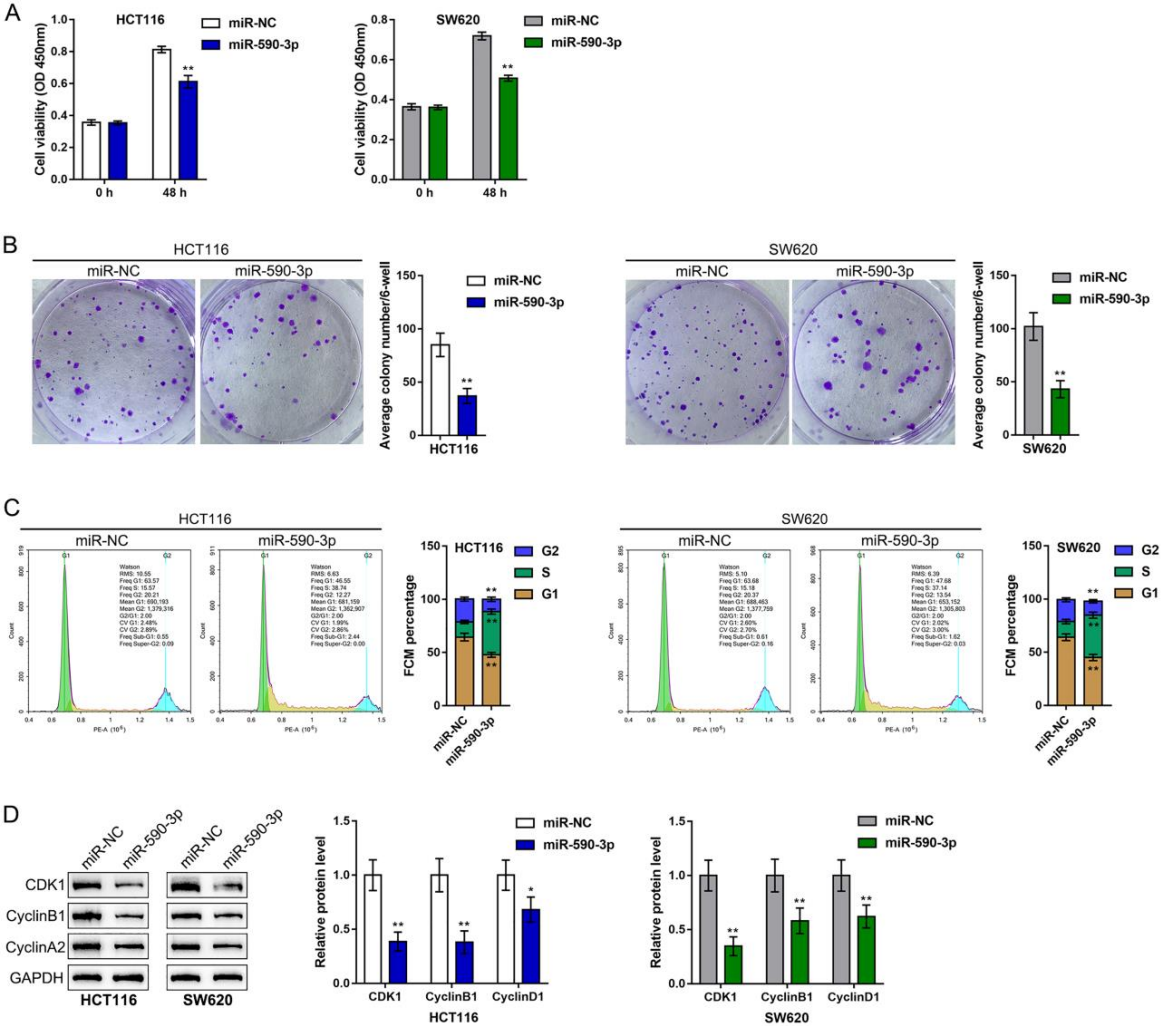
**Effects of miR-590-3p on CRC cell proliferation and cell cycle progression.** HCT116 and SW620 cells were transfected with miR-590-3p, and (**A**) cell viability was examined by CCK-8 assay; (**B**) colony formation capacity was examined by colony formation assay; (**C**) cell cycle progression was examined by flow cytometry; (**D**) the protein levels of CDK1, cyclin B1, and cyclin A2 were examined by immunoblotting. ^**^P < 0.01, compared to the miR-NC group.

After confirming the binding of miR-590-3p to SNHG4 and the CDK1 3'-UTR, this study continued to validate the specific roles of miR-590-3p in the phenotype of CRC cells. We transfected the HCT116 and SW620 cell lines with miR-590-3p and examined related indexes. Similar to SNHG4 knockdown, miR-590-3p overexpression remarkably suppressed cell viability (Figure 5A) and colony formation capacity (Figure 5B). Regarding the cell cycle, miR-590-3p overexpression also induced S phase cell cycle arrest (Figure 5C) and significantly decreased the CDK1, cyclin B1, and cyclin A2 protein levels (Figure 5D). In summary, miR-590-3p can suppress proliferation and induce cell cycle arrest of CRC cells.

### Dynamic effects of SNHG4 and its target miR-590-3p on CDK1 and CRC cell phenotype

**Figure 6:**
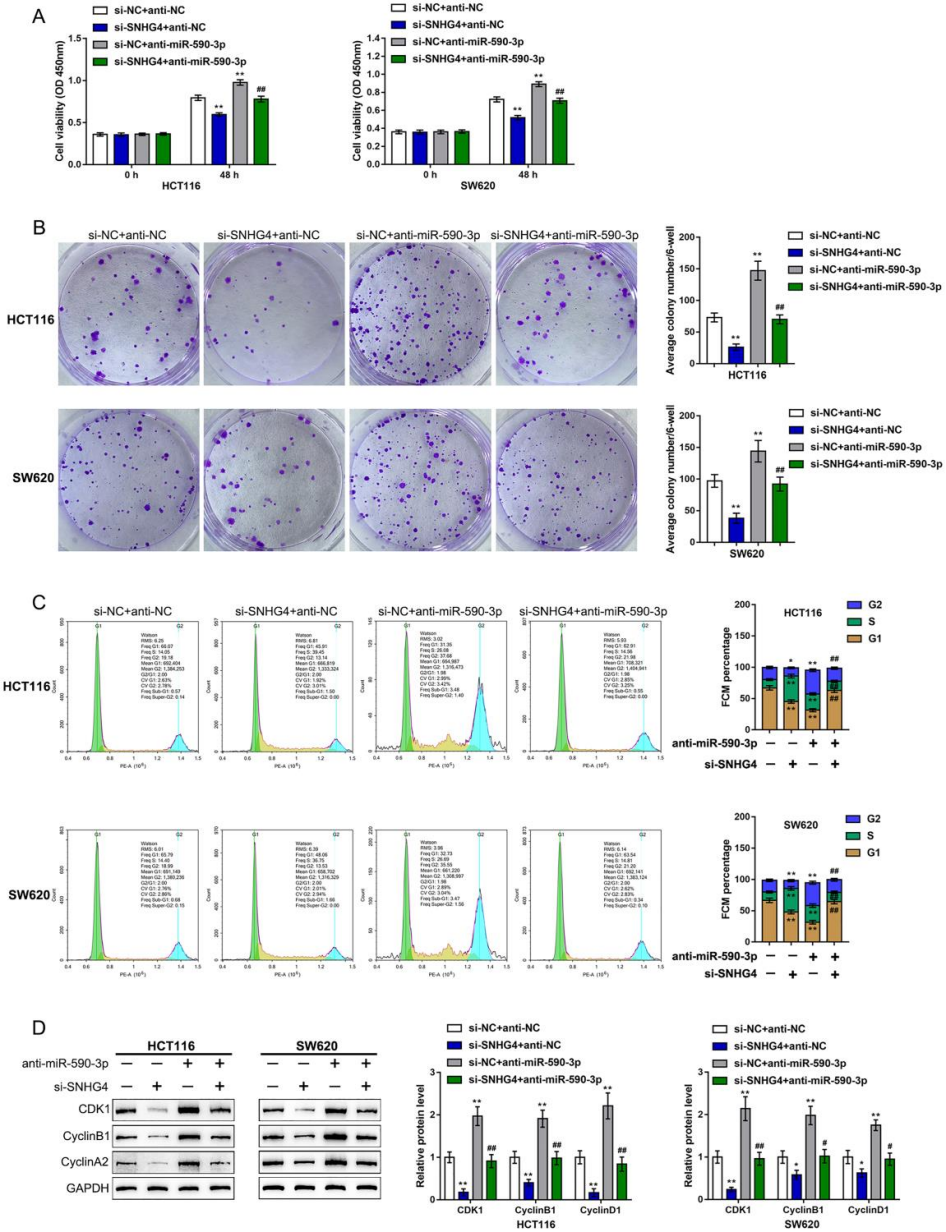
**Dynamic effects of SNHG4 and its target miR-590-3p on CDK1 and CRC cell phenotype.** HCT116 and SW620 cells were cotransfected with si-SNHG4 and anti-miR-590-3p, and (**A**) cell viability was examined by CCK-8 assay; (**B**) colony formation capacity was examined by colony formation assay; (**C**) cell cycle progression was examined by flow cytometry; and (**D**) the protein levels of CDK1, cyclin B1, and cyclin A2 were examined by immunoblotting. ^*^P < 0.05, ^**^P < 0.01, compared to the control group; ^#^*P* < 0.05, ^##^*P* < 0.01, compared to the si-NC (negative control) + anti-miR-590-3p group.

Since miR-590-3p binds to both SNHG4 and the CDK1 3'-UTR, we next investigated whether SNHG4 effects CDK1 expression and the CRC cell phenotype through miR-590-3p. We cotransfected HCT116 and SW620 cells with si-SNHG4 and anti-miR-590-3p and assessed related indexes. SNHG4 knockdown significantly inhibited, while miR-590-3p inhibition promoted, HCT116 and SW620 cell viability and colony formation ability; however, miR-590-3p inhibition dramatically attenuated the effects of SNHG4 knockdown (Figure 6A, 6B).

Regarding the cell cycle, SNHG4 knockdown caused S phase cell cycle arrest, while miR-590-3p inhibition upregulated the proportion of cells in the G2 phase (Figure 6C). Consistently, SNHG4 knockdown suppressed, while miR-590-3p inhibition enhanced, the CDK1, cyclin B1, and cyclin A2 protein levels (Figure 6D). miR-590-3p inhibition significantly attenuated the effects of SNHG4 knockdown on the cell cycle and cell cycle regulators (Figure 6C–6D). In summary, SNHG4 relieves the miR-590-3p-induced inhibition of CDK1 by acting as a ceRNA, thus affecting the CRC cell cycle and cell proliferation.

**Figure 7:**
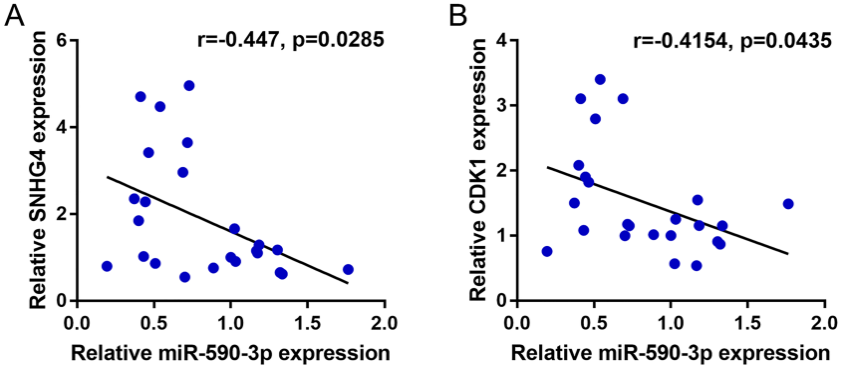
**Correlation of miR-590-3p expression with SNHG4 and CDK1 expression.** (**A–B**) The correlations between miR-590-3p and SNHG4 and between miR-590-3p and CDK1 was determined using Pearson’s correlation analysis.

To further confirm the existence of the SNHG4/miR-590-3p/CDK1 axis in CRC, this study finally analyzed the correlation of miR-590-3p with SNHG4 or CDK1. (Figure 7A–7B) shows that miR-590-3p was negatively correlated with SNHG4 and CDK1. Thus, in CRC, SNHG4/miR-590-3p modulates the cell cycle and cell proliferation through the central cell cycle factor CDK1.

### *In vivo* effects of the SNHG4/miR-590-3p axis on subcutaneously transplanted tumor growth

**Figure 8:**
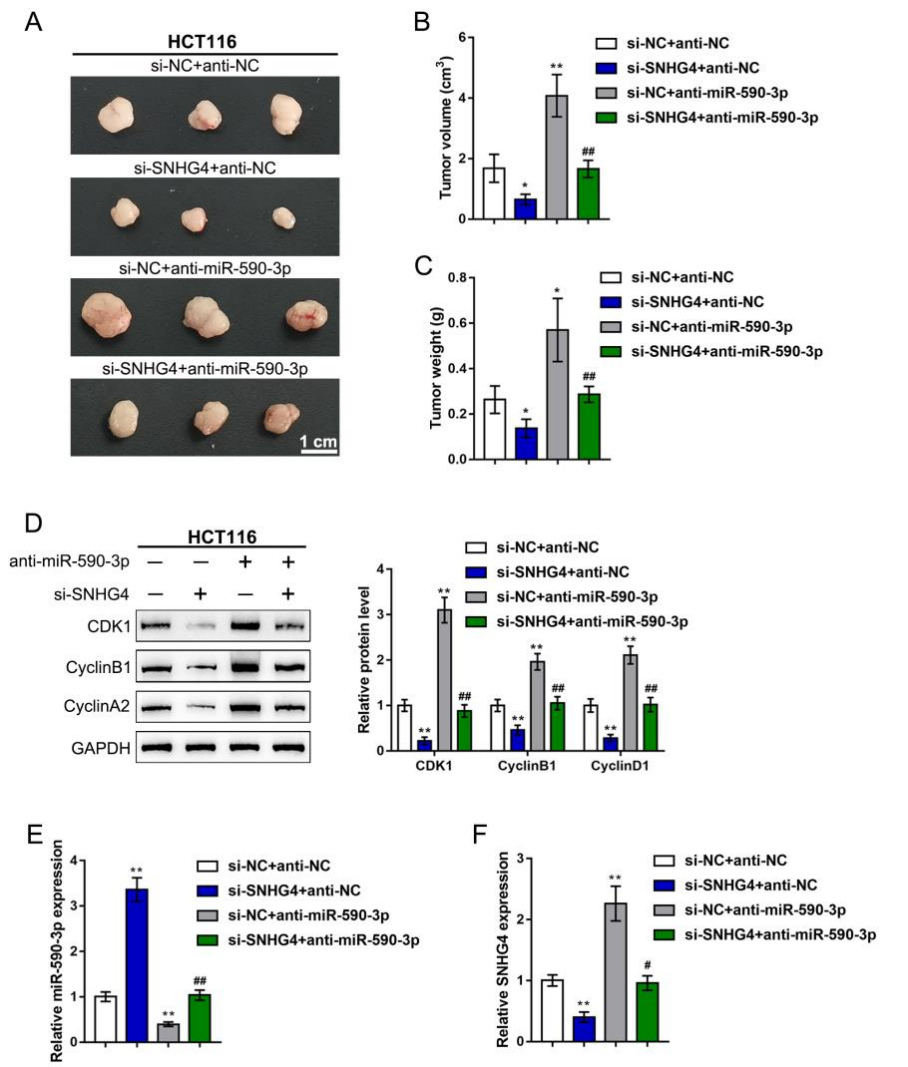
***In vivo* effects of the SNHG4/miR-590-3p axis on subcutaneously transplanted tumor growth.** A subcutaneous transplantation tumor model was established in nude mice, and the mice were divided into four groups: si-NC + anti-NC group, si-SNHG4 + anti-NC group, si-NC + anti-miR-590-3p group, and si-SNHG4 + anti-miR-590-3p group. The mice received subcutaneous injections according to their group. (**A–C**) At day 35 of injection, the tumor size (A) was measured, the tumor volume (B) was calculated, and the tumor weight (C) was measured. (**D**) The protein levels of CDK1, cyclinB1, and cyclinA2 in the tumor samples were determined by immunoblotting. (**E**) The expression level of miR-590-3p was determined by PCR assay. (**F**) The expression level of SNHG4 was detected by PCR assay. ^*^P < 0.05, ^**^P < 0.01, compared to the si-NC + anti-NC group; ^##^*P* < 0.01, compared to si-NC + anti-miR-590-3p group.

Finally, to further confirm the *in vitro* findings, we established a subcutaneous transplantation tumor model in nude mice. The mice were divided into four groups: the si-NC + anti-NC group, si-SNHG4 + anti-NC group, si-NC + anti-miR-590-3p group, and si-SNHG4 + anti-miR-590-3p group. The mice received subcutaneous injections according to their groups. At day 35 of injection, the mice were sacrificed, and the tumors were harvested. As shown in (Figure 8A–8C), SNHG4 silencing reduced, whereas miR-590-3p inhibition increased, the tumor volume and tumor weight; the effects of SNHG4 silencing on tumor growth were significantly reversed by miR-590-3p inhibition. Regarding cell cycle markers, SNHG4 silencing increased, whereas miR-590-3p inhibition decreased, the protein levels of CDK1, cyclinB1, and cyclinA2 in the tumor samples; the effects of SNHG4 silencing on cell cycle markers were also significantly reversed by miR-590-3p inhibition (Figure 8D). In addition, the effect of SNHG4 on miR-590-3p expression was also determined in the mouse tumors. SNHG4 silencing increased, whereas miR-590-3p inhibition decreased, the expression level of miR-590-3p (Figure 8E). Additionally, the expression of SNHG4 was validated in the mouse tissues (Figure 8F). SNHG4 knockdown notably decreased, whereas miR-590-3p inhibition increased, the expression level of SNHG4.

## DISCUSSION

Herein, we found that lncRNA SNHG4 was dramatically increased in CRC tissue samples and cell lines based on both publicly available and experimental data. SNHG4 knockdown suppressed the viability and colony formation capacity of CRC cells. The expression of CDK1 was considerably increased in CRC tissue samples and cells and was positively correlated with the expression of SNHG4 in CRC. SNHG4 silencing not only led to S phase cell cycle arrest but also significantly downregulated the CDK1, cyclin B1, and cyclin A2 protein levels in CRC cells. As predicted by bioinformatics analyses and experimental data, miR-590-3p simultaneously bound to SNHG4 and CDK1. miR-590-3p targeted CDK1 to inhibit its expression. miR-590-3p overexpression exerted the same effects on the CRC cell phenotype as SNHG4 knockdown. When cotransfected into CRC cells, anti-miR-590-3p significantly reversed the effects of si-SNHG4 on CRC cells, indicating that SNHG4 alleviated the miR-590-3p-induced inhibition of CDK1 by acting as a ceRNA. *In vivo*, SNHG4 silencing inhibited subcutaneously transplanted tumor growth and decreased cell cycle marker levels, whereas miR-590-3p inhibition exerted the opposite effects. The *in vivo* effects of SNHG4 silencing were also reversed by miR-590-3p inhibition.

SNHG4 is a SNHG (small nucleolar RNA host gene) [29]. LncRNA SNHG4 has only recently been shown to play a carcinogenic role in other tumors. In osteosarcoma, SNHG4 expression was remarkably elevated, and high SNHG4 expression was correlated with larger tumor size and impaired prognosis of osteosarcoma patients; regarding the molecular mechanism, SNHG4 exerted oncogenic effects in osteosarcoma by sponging miR-224-3p [19]. Moreover, SNHG4 expression was shown to be dramatically upregulated in hepatocellular carcinoma, and SNHG4 upregulation independently predicted lower overall survival of hepatocellular carcinoma patients [30]. Several publicly available microarray expression profiles, including three sets of GEO expression profiles included in the present study, namely, GSE8671, GSE74602, and TCGA-CRC, indicate a similar SNHG4 expression pattern in CRC. Notably, SNHG4 expression was higher in patients with advanced stage CRC, further suggesting that higher SNHG4 expression might be correlated with CRC progression. Herein, we also observed that SNHG4 expression was significantly upregulated in CRC tissues and cells, suggesting a potential oncogenic function of SNHG4 in CRC.

To address the specific effect of SNHG4 in CRC, the present study next knocked down SNHG4 in CRC cell lines with high basal SNHG4 expression levels, namely, HCT116 and SW620 cells. As expected, SNHG4 silencing obviously inhibited HCT116 and SW620 cell viability and colony formation capacity, indicating that SNHG4 knockdown suppressed CRC cell proliferation. Controlling cell proliferation is critical for preventing cancer because cell proliferation exerts important effects on the development of cancers, including the initiation and promotion of tumors [31]. Although the deregulation and control of cancer cell proliferation are complicated, controlling cell proliferation is largely dependent on mechanisms that accelerate or disrupt the cell cycle. Tumor suppressor pathways have been reported to serve as negative signals in transduction systems, leading to cell cycle arrest at different checkpoints [32]. Notably, based on publicly available expression profiles and KEGG and GO analyses, the differentially expressed genes that were positively correlated with SNHG4 were significantly enriched in DNA replication, mismatch repair, and cell cycle pathways, suggesting that SNHG4 might exert its effects through interactions with factors in cell cycle pathways. Thus, CDK1, one of the cyclin-dependent kinases that phosphorylates select proteins to drive cell cycle progression [31], attracted our attention.

The pivotal role of the CDK1/cyclin B1 (CCNB1) complex in the CRC cell cycle has been well described. This complex phosphorylates corresponding substrates, promoting cell cycle transition from the G2 phase to the M phase, namely, the mitosis phase [33, 34]. Fang et al. [35] reported that overexpression of CCNB1 in two CRC cell lines, the HCT116 and SW480 cell lines, induced cancer cell hyperproliferation and local tumor growth; in contrast, CCNB1 knockdown in the same cell lines exerted opposite effects by regulating CDK1 to inhibit cell cycle progression. Another group indicated that lncRNA ZFAS1 directly interacted with CDK1 and observed clear G1 cell cycle arrest due to ZFAS1 knockdown in CRC cells [36]. In the present study, in CRC cells in which SNHG4 was knocked down, we also observed S phase cell cycle arrest, as well as decreased CDK1, cyclin B1, and cyclin A2 protein levels, indicating that SNHG4 exerted its oncogenic effects on CRC cells by affecting the CDK1 levels and the cell cycle.

Mechanistically, studies have provided compelling evidence implicating lncRNAs in transcriptional and posttranscriptional gene expression modulation, genomic imprinting, protein activity regulation, subcellular localization, cell structure maintenance and other different and substantial biological processes [37]. In recent decades, accumulating evidence has revealed that lncRNAs function as competing endogenous RNAs (ceRNAs) and regulate cell growth by sponging miRNAs [27, 28]. In prostate cancer, SNHG4 relieved the miR-377-induced suppression of ZIC5 by acting as a ceRNA, therefore promoting ZIC5-mediated growth and metastasis [20]. In cervical cancer, SNHG4 serves as a ceRNA for miR-148a-3p, thereby upregulating the expression of the miR-148a-3p downstream target c-Met and ultimately promoting the development of cervical cancer [21]. Herein, both bioinformatics and experimental analyses revealed an interaction among SNHG4, miR-590-3p, and CDK1. miR-590-3p overexpression dramatically inhibited CRC cell proliferation, caused S phase cell cycle arrest, and decreased CDK1, cyclin B1, and cyclin A2 protein levels. More importantly, miR-590-3p inhibition exerted opposite effects and significantly reversed the effects of SNHG4 knockdown on the protein level of CDK1 and the phenotype of CRC cells. In summary, SNHG4 also relieved the miR-590-3p-induced inhibition of CDK1 by acting as a ceRNA, thus promoting CRC cell proliferation by affecting the cell cycle.

As a further confirmation of the *in vitro* findings, SNHG4 silencing in subcutaneously transplanted tumors inhibited tumor growth by decreasing cell cycle regulators, whereas miR-590-3p inhibition exerted the opposite effects. Similarly, the *in vivo* effects of SNHG4 silencing were reversed by miR-590-3p inhibition.

## CONCLUSIONS

In conclusion, we demonstrate an axis composed of lncRNA SNHG4, miR-590-3p and CDK1, which influences the cell cycle, ultimately modulating CRC cell proliferation *in vitro* and *in vivo*.

## MATERIALS AND METHODS

### Tissue sampling

A total of 12 human CRC tissues and 12 normal noncancerous tissues were collected from Xiangya Hospital with the approval of the Research Ethics Committee of Xiangya Hospital. Written informed consent was obtained from all of the patients. The enrolled patients were pathologically and clinically diagnosed with colorectal cancer. No radiotherapy or chemotherapy was performed before the surgery. All the fresh tissue samples were immediately stored at -80°C before use in further experiments.

### Cell lines

The fetal colon cell line FHC was obtained from ATCC (CRL-1831™; Manassas, VA, USA) and cultured in DMEM:F12 medium (30-2006; ATCC) supplemented with 10% FBS (Invitrogen, Carlsbad, CA, USA). The CRC cell line HCT8 (CCL-244™) was obtained from ATCC and cultured in RPMI-1640 medium (30-2001; ATCC) supplemented with 10% FBS (Invitrogen). The CRC cell line LoVo (CCL-229™) was obtained from ATCC and cultured in F-12K medium (30-2004; ATCC) supplemented with 10% FBS (Invitrogen). The colorectal cancer cell line HCT116 (CCL-247™) was obtained from ATCC and cultured in McCoy's 5a medium (modified; 30-2007; ATCC) supplemented with 10% FBS (Invitrogen). The colorectal cancer cell line SW620 (CCL-227™) was obtained from ATCC and cultured in Leibovitz's L-15 medium (30-2008; ATCC) supplemented with 10% FBS (Invitrogen). The colorectal cancer cell line HT29 (HTB-38™) was obtained from ATCC and cultured in McCoy's 5a medium (modified; 30-2007; ATCC) supplemented with 10% FBS (Invitrogen). All the cells were cultured at 37°C in 5% CO_2_.

### Expression determination by polymerase chain reaction (PCR)-based analysis

Total RNA was extracted from transfected and/or treated cells with TRIzol reagent (Invitrogen) and treated with DNase I (Invitrogen), following the protocols. Next, oligo (dT) 20 and Superscript II reverse transcriptase (Invitrogen) were used for the synthesis of first-strand cDNA. Finally, the expression of mRNAs and miRNAs was detected using SYBR Green PCR Master Mix (Qiagen), and GAPDH (for mRNA expression) or RNU6B (for miRNA expression) were used as the endogenous controls. The relative expression levels were calculated using the 2^-ΔΔCT^ method. The primers are listed in Supplementary Table 3.

### Cell transfection

The exogenous overexpression or inhibition of miR-590-3p was achieved by transfection of miR-590-3p or anti-miR-590-3p inhibitor (GenePharma, Shanghai, China). SNHG4 knockdown was achieved by the transfection of si-SNHG4 1/2/3 (GenePharma). CDK1 overexpression was achieved by the transfection of the pcDNA3.1- CDK1 plasmid vector (GenePharma). All the transfections were performed using the transfection agent Lipofectamine 3000 (Invitrogen). The sequences of the miR-590-3p mimics, inhibitor and small interfering RNA targeting SNHG4 are listed in Supplementary Table 3.

### Cell viability determination by cell counting kit-8 (CCK-8) analysis

Transfected or non-transfected cells (1×10^4^ cells/ml) were seeded in 96-well cell culture plates and incubated for 24 h. Then, 10 μl CCK-8 agent (03285; Merck, St. Louis, MI, USA) was added to each well followed by another 2-h incubation at 37°C. Next, the optical density (OD value) was determined at a wavelength of 450 nm in a microplate reader.

### Colony formation

Transfected or non-transfected CRC cells were seeded in 6-well plates at a density of 1 × 10^3^ cells/ml and grown into colonies for approximately 14 days. The colonies that formed were fixed with 4% PFA for 30 min. Then, the colonies were stained with 0.1% crystal violet for counting and photographing.

### Immunoblotting analysis

Total proteins were extracted from transfected or non-transfected cells, loaded (50 μg per lane) on 10% sodium dodecyl sulfate (SDS)-polyacrylamide gels, and transferred onto PVDF membranes (Thermo Fisher Scientific). The membranes were blocked for 2 h at 37°C with 5% nonfat milk in Tris-buffered saline with Tween 20 (TBST) and then incubated overnight at 4°C with the following primary antibodies: CDK1 (ab133327, Abcam, Cambridge, UK), cyclin B1 (ab18250; Abcam), cyclin A2 (18202-1-AP; Proteintech, Wuhan, China), MLH1 (ab92312; Abcam), PMS2 (ab110638; Abcam), MSH2 (ab92473; Abcam), MSH6 (ab92471; Abcam) and GAPDH (T0004; Affinity, Changzhou, China). Then, the membranes were incubated with an HRP-conjugated secondary antibody for 1 h at 37°C and covered with ECL luminescence reagent (Perkin-Elmer Inc., Waltham, MA, USA). GAPDH was used as the internal control.

### Cell cycle analysis by flow cytometry

Transfected or non-transfected CRC cells were harvested and fixed with 70% ice-cold ethanol overnight at –20°C. Then, the cells were washed and incubated with RNase A (20 μg/mL in PBS) and PI (50 μg/mL) (Sigma-Aldrich, St. Louis, MO, USA) for 30 min at 37°C in the dark. Cell cycle was analyzed by flow cytometry (Cat. #FC500, Beckman, USA), following previously described methods [38].

### Transwell assay

Transwell compartments with 8-μm pores (Millipore, Billerica, MA, USA) were used to assess the invasive capacity of CRC cells in 24-well plates. A total of 4 × 10^5^ HCT116 and SW620 cells were suspended in medium without serum and were placed in the upper chamber on the coated membrane, and the lower chamber was filled with medium with 10% FBS to stimulate the cells to pass through the membrane. After incubation for 24 h, the cells were stained with 0.1% crystal violet, and the number of invaded cells was calculated from 5 different microscopic fields of view.

### Luciferase reporter assay

Fragments of SNHG4 and the CDK1 3'-UTR were amplified by PCR, introduced into the downstream region of the Renilla psiCHECK2 vector (Promega, Madison, WI, USA), and named wt-SNHG4 and wt-CDK1 3'-UTR, respectively. The seed region of SNHG4 or the CDK1 3'-UTR containing the predicted miR-590-3p-binding site was mutated to construct the mutant vector (mut-CDK1 3'-UTR). These reporter vectors were then cotransfected into 293T cells with miR-590-3p or anti-miR-590-3p, and the luciferase activity was determined 48 h after transfection using the Dual-Luciferase Reporter Assay System (Promega). The Renilla luciferase activity was normalized to the firefly luciferase activity in each transfected well. The primers for plasmid construction are listed in Supplementary Table 3.

### Subcutaneous transplantation tumor model in BALB/C nude mice

HCT116 cells were infected with lentivirus containing negative control small interfering RNA (si-NC) or small interfering RNA targeting SNHG4 (si-SNHG4) and lentivirus containing anti-NC or lentivirus containing anti-miR-590-3p (GeneChem, China). Forty-eight hours later, the infected cells were harvested for subcutaneous injection. Subcutaneous injection was performed following the previously described guidelines [39, 40] using BALB/c nude mice (7 weeks old; SLAC Laboratory Animal Co., Ltd.; Changsha, China). HCT116 cells (1 × 10^7^ cells in 200 μl Matrigel solution) were injected under the skin of the left flank. At day 35, the tumor size was measured, all the animals were sacrificed, and the tumor tissues were collected to examine the protein levels of CDK1, cyclin B1, and cyclin A2 by immunoblotting.

### Data processing and statistical analysis

Data processing and analyses were performed using GraphPad software, and data from three independent experiments are presented as the mean ± S.D. Student’s t-test was used for statistical comparisons between groups. Differences among more than two groups in the above assays were estimated using one-way ANOVA. *P* < 0.05 was considered statistically significant.

### Ethical approval and consent to participate

All procedures performed in studies involving human participants were in accordance with the ethical standards of Xiangya Hospital and with the 1964 Helsinki declaration. Informed consent to participate in the study has been obtained from participants.

### Data availability statement

The authors confirm that the data supporting the findings of this study are available within the article.

## Supplementary Material

Supplementary Figures

Supplementary Tables
