# Supplementary material for: lncRNA SNHG4 modulates colorectal cancer cell cycle and cell proliferation through regulating miR-590-3p/CDK1 axis
Source: Aging (Albany NY). 2021 Mar 19;13(7):9838–58. doi: 10.18632/aging.202737 (PMC8064176; doi:10.18632/aging.202737)
Supplement: Supplementary Figures [file aging-13-202737-s001.pdf]

SUPPLEMENTARY FIGURES

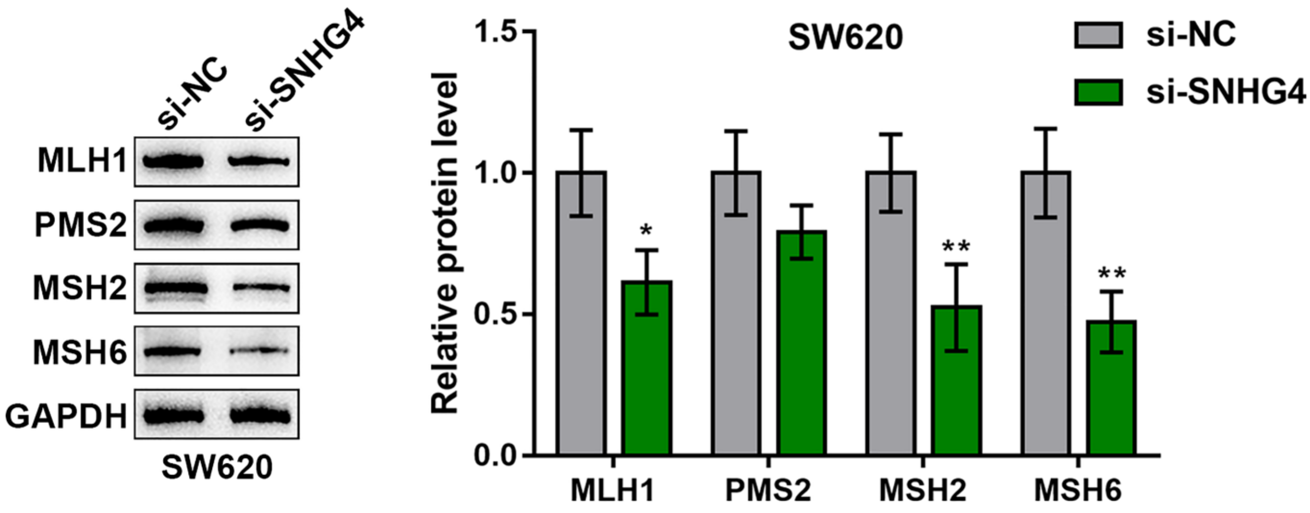

Supplementary Figure 1. Effects of SNHG4 on CRC cell microsatellite stability. SW620 cells were transfected with si-SNHG4, and microsatellite stability-related proteins (MLH1, PMS2, MSH2, and MSH6) were detected by immunoblotting assay. $^{*}P < 0.05$, $^{**}P < 0.01$, compared to si-NC group.

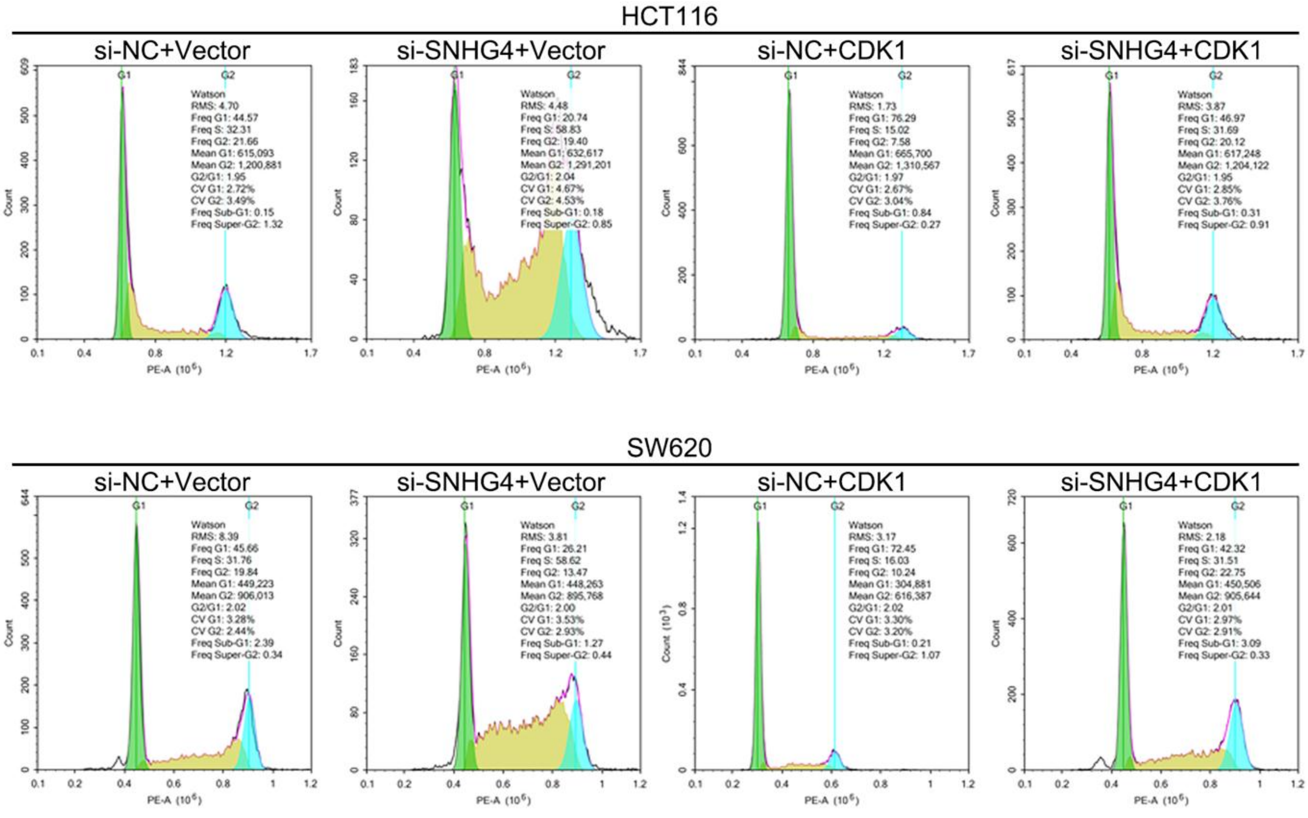

Supplementary Figure 2. The effect of SNHG4 and CDK1 on the CRC cell cycle. HCT116 and SW620 cells were transfected with si-SNHG4 and/or CDK1 overexpression vector, and cell cycle progression was examined by flow cytometry.
