# Supplementary material for: lncRNA SNHG4 modulates colorectal cancer cell cycle and cell proliferation through regulating miR-590-3p/CDK1 axis
Source: Aging (Albany NY). 2021 Mar 19;13(7):9838–58. doi: 10.18632/aging.202737 (PMC8064176; doi:10.18632/aging.202737)
Supplement: Supplementary Tables [file aging-13-202737-s002.pdf]

## SUPPLEMENTARY TABLES

**Supplementary Table 1. KEGG pathway annotation on 44 SNHG4-related genes.**

| #term ID | term description | observed gene count | background gene count | false discovery rate | matching proteins in your network (IDs) | matching proteins in your network (labels) |
|---|---|---|---|---|---|---|
| hsa03030 | DNA replication | 2 | 36 | 0 | ENSP00000264156, ENSP00000369411 | MCM6,RFC3 |
| hsa03430 | Mismatch repair | 2 | 23 | 0 | ENSP00000355506, ENSP00000369411 | EXO1,RFC3 |
| hsa04110 | Cell cycle | 3 | ## | 0 | ENSP00000264156, ENSP00000378699, ENSP00000405726 | CDC45,CDK1,MCM6 |

**Supplementary Table 2. GO functional analysis on 44 SNHG4-related genes.**

| #term ID | term description | observed gene count | background gene count | false discovery rate | matching proteins in your network (IDs) | matching proteins in your network (labels) |
|---|---|---|---|---|---|---|
| GO:0003697 | single-stranded DNA binding | 4 | 99 | 0 | ENSP00000264156, ENSP00000309595, ENSP00000405726, ENSP00000418268 | C10orf2,CDC45,MCM10, MCM6 |
| GO:0140097 | catalytic activity, acting on DNA | 5 | ## | 0 | ENSP00000264156, ENSP00000309595, ENSP00000328835, ENSP00000355506, ENSP00000405726 | C10orf2,CDC45,EXO1,MCM6, TOP1MT |
| GO:0003678 | DNA helicase activity | 3 | 41 | 0 | ENSP00000264156, ENSP00000309595, ENSP00000405726 | C10orf2,CDC45,MCM6 |
| GO:0017111 | nucleoside-triphosphatase activity | 8 | ## | 0 | ENSP00000260363, ENSP00000264156, ENSP00000309595, ENSP00000356319, ENSP00000369411, ENSP00000377601, ENSP00000378356, ENSP00000405726 | C10orf2,CDC45,HELLS, KIF14, KIF20A,KIF23,MCM6,RFC3 |
| GO:0004386 | helicase activity | 4 | ## | 0 | ENSP00000264156, ENSP00000309595, ENSP00000377601, ENSP00000405726 | C10orf2,CDC45,HELLS, MCM6 |
| GO:0003688 | DNA replication origin binding | 2 | 14 | 0 | ENSP00000405726, ENSP00000418268 | CDC45,MCM10 |
| GO:0008144 | drug binding | 11 | ## | 0 | ENSP00000260363, ENSP00000264156, ENSP00000309595, ENSP00000348838, ENSP00000355966, | C10orf2,CDK1,HELLS,KIF14, KIF20A,KIF23,MCM6,NEK2, NOLC1,PPIL1,UBE2C |

| | | | | | | |
|---|---|---|---|---|---|---|
| | | | | | ENSP00000356319, ENSP00000362803, ENSP00000377601, ENSP00000378356, ENSP00000378699, ENSP00000385410 | |
| GO:0005524 | ATP binding | 10 | ## | 0 | ENSP00000260363, ENSP00000264156, ENSP00000309595, ENSP00000348838, ENSP00000355966, ENSP00000356319, ENSP00000377601, ENSP00000378356, ENSP00000378699, ENSP00000385410 | C10orf2,CDK1,HELLS,KIF14, KIF20A,KIF23,MCM6,NEK2, NOLC1,UBE2C |
| GO:0003777 | microtubule motor activity | 3 | ## | 0 | ENSP00000260363, ENSP00000356319, ENSP00000378356 | KIF14,KIF20A,KIF23 |
| GO:0008139 | nuclear localization sequence binding | 2 | 28 | 0 | ENSP00000346453, ENSP00000385410 | IPO4,NOLC1 |
| GO:1901363 | heterocyclic compound binding | 20 | ## | 0 | ENSP00000240488, ENSP00000260363, ENSP00000264156, ENSP00000309595, ENSP00000322180, ENSP00000328835, ENSP00000331879, ENSP00000348838, ENSP00000355506, ENSP00000355925, ENSP00000355966, ENSP00000356319, ENSP00000369411, ENSP00000377601, ENSP00000378356, ENSP00000378699, ENSP00000385410, ENSP00000405726, ENSP00000418268, ENSP00000443647 | AUNIP,C10orf2,CDC45, CDK1, DSCC1,EXO1,HELLS,KIF14,KIF20A, KIF23,MCM10,MCM6,MND1,NEK2, NOLC1,PRMT3,PROX1, RFC3, TOP1MT,UBE2C |
| GO:0097159 | organic cyclic compound binding | 20 | ## | 0 | ENSP00000240488, ENSP00000260363, ENSP00000264156, ENSP00000309595, ENSP00000322180, ENSP00000328835, ENSP00000331879, ENSP00000348838, ENSP00000355506, ENSP00000355925, ENSP00000355966, | AUNIP,C10orf2,CDC45, CDK1, DSCC1,EXO1,HELLS,KIF14, KIF20A,KIF23,MCM10, MCM6, MND1,NEK2,NOLC1,PRMT3, PROX1,RFC3,TOP1MT, UBE2C |

| | | | | | | |
|---|---|---|---|---|---|---|
| | | | | | ENSP00000356319, ENSP00000369411, ENSP00000377601, ENSP00000378356, ENSP00000378699, ENSP00000385410, ENSP00000405726, ENSP00000418268, ENSP00000443647 | |
| GO:0003677 | DNA binding | 12 | ## | 0 | ENSP00000240488, ENSP00000264156, ENSP00000309595, ENSP00000322180, ENSP00000328835, ENSP00000355506 ,ENSP00000355925, ENSP00000369411, ENSP00000385410, ENSP00000405726, ENSP00000418268, ENSP00000443647 | AUNIP,C10orf2,CDC45, DSCC1, EXO1,MCM10,MCM6,MND1, NOLC1,PROX1,RFC3,TOP1MT |
| GO:0019899 | enzyme binding | 11 | ## | 0 | ENSP00000265295, ENSP00000309595, ENSP00000314897, ENSP00000346453, ENSP00000348838, ENSP00000355966, ENSP00000356319, ENSP00000376457, ENSP00000378356, ENSP00000385410, ENSP00000418268 | ANGPT2,C10orf2,ECT2,IPO4,KIF14, KIF20A,MCM10,NEK2, NOLC1, SPDL1,UBE2C |
| GO:0097367 | carbohydrate derivative binding | 11 | ## | 0 | ENSP00000260363, ENSP00000264156, ENSP00000309595, ENSP00000348838, ENSP00000355966, ENSP00000356319, ENSP00000377492, ENSP00000377601, ENSP00000378356, ENSP00000378699, ENSP00000385410 | C10orf2,CDK1,HELLS,HMMR, KIF14,KIF20A,KIF23,MCM6, NEK2,NOLC1,UBE2C |

**Supplementary Table 3. The primer sequence.**

| Name | Forward | Reverse |
|---|---|---|
| QPCR-SNHG4 | ACCGAAGCCACGCCCAGTA | TCACCTGCCACTATTTCCTCCC |
| QPCR-GAPDH | ACAGCCTCAAGATCATCAGC | GGTCATGAGTCCTTCCACGAT |
| QPCR-CDK1 | AAACTACAGGTCAAGTGGTAGCC | TCCTGCATAAGCACATCCTGA |
| QPCR-miR-590-3p | RT:GTCGTATCCAGTGCGTGTCGTGGAGTCGGCAATTGCACTGGATACGACACTAGC<br>F: GCCGGCCTAATTTTATGTATAA | CAGTGCGTGTCGTGGA |
| QPCR-U6 | CTCGCTTCGGCAGCACA | AACGCTTCACGAATTTGCGT |
| Si-NC | UUCUCCGAACGUGUCACGUTT | ACGUGACACGUUCGGAGAATT |
| Si-SNHG4 1# | UAUUUCCUCCCUUCAGAUGTT | CAUCUGAAGGGAGGAAAUATT |
| Si-SNHG4 2# | ACUACAUACGUUACUUAUCTT | GAUAAGUAACGUAUGUAGUTT |
| Si-SNHG4 3# | ACAUACGUUACUUAUCUUGTT | CAAGAUAAGUAACGUAUGUTT |
| MiR-NC mimics | UUCUCCGAACGUGUCACGUTT | ACGUGACACGUUCGGAGAATT |
| MiR-590-3p mimics | UAAUUUUAUGUAUAAGCUAGU | UAGCUUAUACAUAAAAUUAUU |
| Inhibitor NC | CAGUACUUUUGUGUAGUACAA | |
| MiR-590-3p inhibitor | ACUAGCUUAUACAUAAAAUUA | |
| Wt-CDK1 3’UTR | aattctaggcgatcgctcgagATGAATTTAAATATAATTCTGTAAATGTGTGTA | attttattgcggccagcggccgcTATTCATCTTTAGCCAGGTTGTATAGTTAA |
| mut-CDK1 3’UTR | GAACTACtttttattAGGAAAATGCTAAGTTCAAGTTTCG | CCTaataaaaaGTAGTTCCAACTACAGGAAAATAAAACTG |
| Wt-SNHG4 | aattctaggcgatcgctcgagAGATGCCTTCACCTGAATGACAT | attttattgcggccagcggccgcTGAGGCAGGAGAATCGCTTG |
| mut-SNHG4 | GGGGACTtttttaaaTGATGTGAAAAATTATGTAGAGTATCAGACT | TCAtttaaaaaAGTCCCCTACCCCCATCTGAGC |
| Lentivirus Anti-NC sequence | CAGTACTTTGTGTAGTACAACGCGCAGTACTTTGTGTAGTACAACGCGCAGTACTTTGTGTAGTACAACGCGCAGTACTTTGTGTAGTACAACGCGCAGTACTTTGTGTAGTACAACGCGCAGTACTTTGTGTAGTACAACGCGCAGTACTTTGTGTAGTACAACGCG | |
| Lentivirus Anti-miR-590-3p sequence | ACTAGCTTACTGTAAAATTACGCGACTAGCTTACTGTAAAATTACGCGACTAGCTTACTGTAAAATTACGCGACTAGCTTACTGTAAAATTACGCGACTAGCTTACTGTAAAATTACGCGACTAGCTTACTGTAAAATTACGCGACTAGCTTACTGTAAAATTACGCG | |
